# Repeatability of Myotonometric Muscle Measurements in Infants Aged 0–3 Months: Toward an Objective Tool Supporting Early Motor Assessment

**DOI:** 10.3390/jcm15103699

**Published:** 2026-05-11

**Authors:** Agnieszka Ptak, Agnieszka Owczarek, Agnieszka Browarska, Rita P. Romero-Galisteo, Manuel González-Sanchez, Małgorzata Stefańska

**Affiliations:** 1 Faculty of Physiotherapy, Wroclaw University of Health and Sport Science, al. Paderewskiego 35, 51-612 Wroclaw, Poland; malgorzata.stefanska@awf.wroc.pl; 2 A. Falkiewicz Specialist Hospital in Wroclaw, 2 Warszawska St., 52-114 Wroclaw, Poland; aowczarek.neonatolog@gmail.com; 3 Children’s Specialists Foundation, 16/32 Chorwacka Street, 51-107 Wroclaw, Poland; kontakt@lekarskimokiem.pl; 4 Departamento de Fisioterapia, Facultad CC de la Salud, Universidad de Málaga, 29071 Málaga, Spain; rpromera@uma.es (R.P.R.-G.); mgsa23@uma.es (M.G.-S.)

**Keywords:** newborns, repeatability, myotonometry, muscles, development, measurement

## Abstract

**Background**: Early detection of developmental disorders in infants is an important topic for scientists and clinicians, but above all for children and their families. Early detection of changes in the mechanical properties of muscles is crucial for starting therapeutic processes. This study aims to fill the research gap regarding the use of myotonometry in the infant patient group (aged 0–3 months). The study aimed to evaluate the test–retest repeatability of myotonometric measurements in infants at three time points, with particular attention to potential age-related differences. **Methods**: The study group consisted of healthy newborns born from physiological pregnancies with an Apgar score of 8–10 points. The studies began on the 1st–3rd day of life and lasted until the 12th week in cycles every 6 weeks. **Results**: In the study conducted on the 1st–3rd day of life, the repeatability of the studied parameters can be described as moderate (muscle tone, elasticity, relaxation, and creep) or poor (stiffness). Measurements conducted in the 6th week of life show high repeatability of muscle stiffness and elasticity or moderate repeatability of muscle tone, creep, and relaxation time. Measurements in the 12th week of life of the infants may be considered as high repeatability of muscle tone, stiffness, relaxation time, and the creep parameter, and moderate muscle elasticity. **Conclusions**: The study demonstrated the usefulness of using myotonometric measurement for infants from the 6th and 12th week of age in the studied parameters, confirmed by the high repeatability of measurements. Whereas in the group of newborns aged 1–3 days, the repeatability of all analyzed parameters can be described as moderate or low.

## 1. Introduction

Newborns constitute a difficult group for the assessment of movement patterns and muscle parameters assessed as muscle tone. Neonatal developmental assessment in clinical settings should be straightforward and accessible for all patients. Any concerns about the assessment due to potential human error should be verified using objective, noninvasive, and user-friendly assessment methods [1,2].

The most described current gold standard assessments of motor development are the General Movements Assessment (GMA) or the Hammersmith Infant Neurological Examination (HINE) [3,4].

Scales usually describe the motor skills demonstrated by the infant by comparing observed functions with described normative patterns. Despite the demanding training and years of experience of specialists, the quality of the motor pattern is often controversial due to the subjective nature of the assessment [5,6,7].

Muscle tone in all newborns is routinely assessed after delivery using the Apgar scale. It is considered one of the early markers of autism [8] and genetic disorders, such as Duchenne dystrophy [9] or hemophilia [10]. Myotonometry has been effectively used in pediatric cases, particularly for children with SMA types I and II. It demonstrated excellent intra- and inter-rater reliability for measuring muscle tone (frequency) and stiffness in the biceps, triceps, rectus femoris, and gastrocnemius, with the ICC values indicating mostly excellent reliability and gastrocnemius stiffness at ICC = 0.71, categorized as “good,” within a single session [11,12]. In a study involving children with developmental disabilities (26 participants), myotonometry was applied to upper and lower limb muscles, showing good to excellent intra- and inter-rater reliability (ICC ≈ 0.68–0.95) and a small measurement error, making it a supportive clinical tool for quick tone assessments [13]. Additionally, in children with cerebral palsy, the MyotonPRO instrument identified increased stiffness in the rectus femoris and hip adductors compared to typically developing peers, with correlations seen between adductor stiffness and the severity of cerebral palsy [14]. However, current research supports a correlation between altered viscoelastic muscle properties and specific genetic diseases, particularly in adults and older children [15,16,17,18]. The early development of viscoelastic muscle parameters in healthy children is still not well known. Although most existing evidence comes from research focused on older age groups, it offers a theoretical basis indicating that early changes in neuromuscular characteristics might also occur in the first few months of life.

In the case of neonatal examinations, an important criterion that the measuring equipment should meet is non-invasiveness and ease of use. Myotonometry represents a portable, non-invasive, and user-friendly diagnostic technology that has the potential to enhance early diagnostic evaluations in infants within routine clinical settings. The myotonometer exemplifies this technology, as it effectively measures the viscoelastic properties of skeletal muscles [19]. It is a measurement tool used to assess the physical characteristics of muscles in healthy people of different ages [15,20] as well as in people burdened with disease processes such as stroke [10], neuromuscular disorders [21], chronic spinal pain [22], and genetic disease [23]. Studies using myotonometry conducted on adults show that this method is valid, reliable, and repeatable in this study group [24,25,26]. Myotonometry is also used to assess changes in muscle parameters that occur as a result of training and therapy [27,28]. The first attempt to use muscle assessment in infants before and after therapy was undertaken in the work of Ptak [29]. In their work, the authors assessed infants aged 4–9 months before and after therapy. Various muscles are used for myotonometry studies [10]. Although there is no universal value for all muscles, lower limb muscles meet the research requirements in terms of muscle size, location, and accessibility [30]. The importance lies in standardizing the procedure during the research [27].

Objectification of the assessment of therapeutic progress is important for clinical trials, diagnostics, programming of the therapeutic process, and observation of changes occurring after the introduction of therapeutic procedures. Early diagnosis allows for proper referral of patients for appropriate intervention. It helps to optimize developmental potential and improve the quality of life of the child and family in the future [31]. In both scientific and clinical research, the reliability of the study is crucial. Objective assessment of the physical characteristics of muscles is possible through the use of measuring equipment [32]. The basic parameter defining the credibility and usefulness of the study is its repeatability. The credibility of the study is the level to which the results obtained in the study are free from random error. If the credibility of the measurements is not verified, the results of the study may be questioned. Consequently, the feature assessed during the test may be falsified by an error made at the level of observation of the variable [33]. Repeatability studies are the foundation for observations used to assess capabilities and functions, and in the application of therapeutic processes. They help avoid measurement errors related to the subjective nature of the assessor [33].

The present study sets out to explore the stabilization of muscle parameters in infants over time, with the inspiring goal of evaluating the repeatability of myotonometric measurements. As elastoplastic muscle properties are known to vary across different clinical conditions. Uncovering developmental changes in early infancy may lay the groundwork for transformative future investigations into their potential diagnostic applicability. The assessment of the possibility of using myotonometry in the muscle assessment of the youngest patients is still little known and requires further research. With our work, we try to fill the gap existing in research.

The study aimed to evaluate the test–retest repeatability of myotonometric measurements in infants at three time points, with particular attention to potential age-related differences.

## 2. Material and Methods

### 2.1. Study Group

The studies were conducted between November 2023 and July 2024 at the Falkiewicz Specialist Hospital in Wrocław in the maternity ward where mothers stay with their newborns. Parents could voluntarily register their participation in the studies.

**Figure 1:**
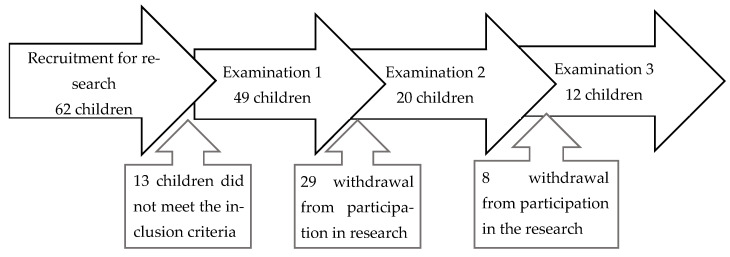
Recruitment and course of the study.

The studies were conducted on 62 infants aged 1–3 days. Study 1 was attended by 49 infants, and study 2 was conducted in the 6th week of life by 20 infants. The studies were completed by 12 infants in the 12th week. The participation of infants in the studies is presented in the flowchart diagram (Figure 1).

Sample size N = 12 was determined using G*Power software 3.1 based on a repeated-measures ANOVA model (within-subject factors). The following assumptions were applied: significance level α = 0.05, effect size *f* = 0.40, statistical power (1 − β) = 0.80, and three repeated measurements within subjects. Neonatal and preterm infant research groups typically retain approximately 60–90% of participants; however, some studies report attrition rates of up to 70% [34]. The study group consisted of healthy newborns with an Apgar score of 8–10 points, born naturally (68%) or by cesarean section (38%), with an average birth weight of 3271 g (±504 g).

Inclusion criteria for the study: physiological course of pregnancy, uncomplicated course of labor and perinatal period, condition of the newborn determined on the Apgar scale at 8–10 points.

Exclusion criteria for the study were premature birth, intrauterine growth disorders, other disease processes, congenital and genetic defects, adaptive disorders of the neonatal period, condition determined on the Apgar scale at 0–7 points.

It is important to note that the final sample of participants who completed all assessment time points (n = 12) represents a subset of the originally recruited cohort rather than a pre-determined sample size. However, the smaller sample size may still limit statistical power, and the findings—especially those from the 12-week follow-up—should be interpreted with caution.

### 2.2. Assessment

The examination was always performed in the right-side-lying position. Myotonometric evaluation was performed on the left calf in the middle part of the gastrocnemius muscle of the lateral head of the muscle. This examination was performed three times at the same point. Measurements were performed in triplicate by the same examiner to assess test–retest repeatability. Measurements were taken using a myotonometer that was set up to deliver three consecutive impulses. The device automatically calculated the average value and provided real-time quality control. Any measurements with excessive variability were flagged and repeated, ensuring that only stable and reliable values were included in the analysis. The patient was calm and relaxed during the examination. The initial examination was conducted within the hospital ward, specifically in the designated room where the neonate resided with the mother under the rooming-in system. Two subsequent measurements were performed in the neonatal ward during a follow-up visit. Throughout the examination process, the infant’s mother maintained proximity to her child, ensuring continual contact and fostering an environment of emotional support. In each instance, the conditions were optimized for comfort; the infant was adequately nourished and attended to, which contributed to a state of calm alertness during the assessments. The gastrocnemius muscle in neonates presents a promising opportunity for myotonometric assessment. Being a superficial muscle located less than 2 cm deep, it facilitates reliable measurements using MyotonPRO Tallinn, Estonia.

Measurements of viscoelastic muscle parameters were carried out using the MYOTON pro myotonometer (Myoton^®^ Tallinn, Estonia) in the side-lying position. The measurement consisted of placing the head of the device perpendicular to the skin over the central part of the tested muscle at rest. The device examines the mechanical, dynamic response of the tissue to the acceleration of oscillations and enables subsequent calculation of parameters characterizing the state of stress, biomechanical, and viscoelastic properties.

The following parameters were recorded:

Oscillation frequency (Hz) (F) (muscle tone) characterizes the internal tension of biological soft tissues at the cellular level. The oscillation frequency characterizes the tension of superficial skeletal muscles in a passive or resting state, without voluntary contraction.

Dynamic stiffness (N/m) (S) characterizes the resistance of biological soft tissues to force deformation.

Elasticity (D) logarithmic decrement (arb) characterizing the damping of tissue vibrations.

The faster the tissue oscillations disappear, the greater the dissipation of the mechanical energy contained in the measurement pulse. The decrease in natural tissue vibrations describes elasticity. Elasticity is a biomechanical property of soft tissues that characterizes the ability to return to its original shape after deformation. The greater the loss, the lower the elasticity. Theoretically, a decrease of zero (0) means absolute flexibility (no damping).

Relaxation (ms) (R) relaxation time of mechanical stresses (ms) characterizing tissue regeneration after displacement. The higher the tension or stiffness of the tissue, the faster the tissue regains its shape, which means a lower value.

Creep (arb) (C) is the ratio of relaxation time and strain characterizing creep, i.e., the gradual elongation of tissue over time under the influence of constant tensile stress.

The higher the structural integrity or stiffness of the tissue, the higher its creep resistance, which means the lower the value.

Myotonometric measurements were performed three times: on the 1st–3rd day of the child’s life and on the 6th and 12th weeks. Each test was consecutively performed three times. Measurements were taken with three bumps until no red flags were detected on the device. Operator experience measurements were performed by one researcher with 5 years of experience using Myoton Pro and 15 years of neurodevelopmental diagnostic experience. Newborns and infants were always placed on their right side. The measurement was performed on the left lower leg on the medial part of the gastrocnemius muscle of the lateral head. The patient was calm and relaxed. The measurement position was dictated by the natural rotation of the head to the right that the child presents after birth [35]. The examination procedure was as follows. The probe was placed perpendicular to the skin’s surface directly on the pre-marked measurement point above the muscle being measured. The device was moved towards the skin until the green light was illuminated to indicate the accurate measurement position. Then the device was gently held and kept steady within the measurement position until the device automatically performed the measurements.

The research was conducted in accordance with the Declaration of Helsinki. The authors obtained informed consent from all participants and/or their legal guardians to participate in the study, and informed consent from a parent and/or legal guardian was taken. Each participant could withdraw from the study at any stage of the project. The study was conducted in accordance with relevant guidelines and regulations with the consent and under the supervision of the Senate Bioethics Committee of Wroclaw University of Health and Sport Science, No. 24/2021. The trials are registered with the New Zealand Clinical Trials Registry No. ACTRN 12622000417785, the first data registered: 11 March 2022.

### 2.3. Statistical Analysis

Before conducting analyses, the Kolmogorov–Smirnov test confirmed the normal distribution of all variables. To characterize the results, the mean was used as a measure of central tendency, and the standard deviation as a measure of dispersion. The analyses were descriptive in nature and focused on repeatability assessment rather than hypothesis testing.

A frequently used measure of measurement repeatability is the intraclass correlation coefficient (ICC). The ICC can be calculated according to model 1 (one-way) or model 2 (bidirectional). Both models provide different information about the reliability of the method. The two-way ICC(A,1) model provides an estimate of reliability when the variable is assessed by the same set of raters, taking into account the effect of bias, whereas the one-way ICC(C,1) model, which requires different raters, ignores this effect [35,36]. The analysis conducted by Liljequist (2019) [36] proves that ICC models 1 and 2 can, contrary to previous opinions, be used interchangeably [37]. In the statistical analysis performed to develop the results of our research, it was decided to use the classical division proposed by Koo & Lii (2016) [35] and Shrout & Fleiss (1979) [38] model 2, type 1 (single rater), with the effect measure in the form of absolute agreement, which was used for the calculations [38,39]. In addition to the ICC value, the 95% confidence interval (CI) for the calculated intraclass correlation coefficient was used as a measure of agreement because the final assessment of the level should be made not based on the ICC value alone, but together with the value of the particularly lower limit of the 95% confidence interval [35,36,39]. Additionally, the Standard Error of Measurement (SEM = SD × √(1 − ICC)) and the Minimal Detectable Change at the 95% confidence level (MDC_95_ = 1.96 × SEM × √2) were calculated.

The ICC value ranges from −1 to 1, where 1 means full agreement of measurements, 0 means agreement of measurements at a random level (no agreement), and negative values of the coefficient indicate a complete lack of agreement [40,41]. In the detailed interpretation, an ICC value of less than 0.50 indicates poor agreement, an ICC value between 0.50 and 0.75—moderate agreement, an ICC value between 0.75 and 0.90—high agreement, and an ICC value above 0.90—excellent agreement [38].

In order to confirm the level of repeatability of the performed myotonometric measurements for all analyzed variables, Bland–Altman plots were generated three times and included in the Supplementary Materials Files S1–S3. Calculations were performed using Statistica 14.1 and PQstat 1.8.4. In all analyses, *p* < 0.05 was considered the level of significance.

This study was reported in accordance with the GRRAS (Guidelines for Reporting Reliability and Agreement Studies) recommendations.

## 3. Results

Three-time examination of the mechanical properties of the triceps calf muscle conducted on the 1st–3rd day of the child’s life showed a variety of results. The largest difference between the highest and lowest measurement of 4.6% was observed for muscle elasticity. The repeatability of all analyzed features can be described as moderate (muscle tone (F), elasticity (D), relaxation (R), and creep (C)) or low (stiffness (S)) (Table 1 and Table 2).

Measurements taken at 6 weeks of age show high repeatability of muscle stiffness and elasticity, moderate muscle tone, creep, and relaxation time. The smallest difference between the highest and lowest measurement was recorded for muscle elasticity (1.3%), the largest, 9.4%, for relaxation time (Table 3 and Table 4).

The agreement of the measurements of the mechanical properties of the triceps calf muscle estimated by the ICC at 12 weeks of age of the infants showed the high repeatability of stiffness, muscle tone, relaxation time, and creep, and moderate muscle elasticity. The smallest difference between the measurements was recorded for muscle tone and relaxation time. It amounted to 0.5% and 2.3%, respectively. The largest percentage difference between the highest and the lowest measurement was observed for elasticity (11.2%) (Table 5 and Table 6).

## 4. Discussion

With the growing awareness of the importance of evidence-based practice, researchers and clinicians are interested in objective assessment of the properties and functions of the body, as well as the effectiveness of the applied treatment and physiotherapy techniques. The basis for assessing the development of infants in the first months of life is the assessment of their motor development [5,42]. Clinical exams of infant motor skills and muscle tone rely heavily on the examiner’s observation and judgment, which can introduce variability and bias. Many standard tools (e.g., Bayley-III, AIMS, TIMP, NSMDA, HINE, MOS-R, IMP) are observational and criterion-based, not instrument-based [43]. Standardized scales of psychometric assessment reduce the level of subjectivity but do not eliminate it and, if used well, demonstrate good to excellent reliability [37,44,45]. The most controversial aspect of muscle tone assessment is its subjective nature and insensitivity to small changes. Muscle tone is most often assessed by palpation, but this test is also subjective [46]. Systematic reviews indicate the need for objective tools for measuring muscle tone and caution in interpreting results obtained using subjective tools [43,47]. Motor development, together with the assessment of the function of the musculoskeletal system, is usually carried out by comparing the child’s condition with developmental norms. This group of tools includes sheets that allow for a qualitative assessment of the pediatric patient. These are easily accessible and non-invasive methods, but due to the subjective nature of the assessment of the examiner, their reliability and repeatability are limited [48,49].

Centuries ago, muscle tone was defined as resistance to passive movement. However, since then, researchers have emphasized the complexity of elastoplastic muscle properties. Muscle tone is a complex and multidimensional concept, encompassing both neural and non-neural components. In addition to the electrical activity recorded by EMG, associated with the stimulation of alpha-motoneurons, it also includes electromyographically silent (EMG-silent) elements resulting from the intrinsic viscoelastic properties of muscle fibers and surrounding connective tissues, including fascia. Mechanical factors, such as actin-myosin bridge interactions, the phenomenon of thixotropy, and passive tissue stiffness, contribute significantly to the resistance felt during testing [50,51,52]. Because palpation assesses the combined effect of these neural and mechanical components, as well as the properties of adjacent soft tissues, the result reflects global myofascial resistance rather than a single physiological parameter. Therefore, clinical assessment of muscle tone by palpation—particularly in infants—should be interpreted as a complex measure, influenced by the interaction of multiple biomechanical and neurophysiological factors [42,53]. Studies on the repeatability of myotonometric measurements have so far been performed mostly in adults. Most studies have shown high and very high repeatability of measurements using the Myoton Pro device. In the study by Pruyn et al. (2016) [24], the repeatability of three methods of measuring muscle stiffness was tested. One of the methods was myotonometric measurement. In a test–retest study performed with an interval of 1 week in 15 female volleyball players, high and excellent agreement of measurements was demonstrated for all analyzed muscles (lateral gastrocnemius, medial gastrocnemius, soleus, achilles aponeurosis) in all analyzed positions: standing (0.83–0.96), lying (ICC 0.72–0.98) and during contraction (0.69–0.93) [10].

Garcia-Bernal et al. (2021) [20] conducted a meta-analysis assessing the validity and reliability of myotonometry in assessing the viscoelastic properties of muscles in patients after stroke, assessing the validity and reliability of myotonometry measurements in the case of stroke as a complementary measurement tool. Most of the reviewed studies showed moderate to very high intra-rater reproducibility for both upper (0.72 to 0.96) and lower limbs (ICC = 0.62 to 0.92). Single-study inter-rater reliability showed moderate to very high reliability for upper limbs (ICC = 0.65 to 0.93) and ICC = 0.65 to 0.99 for lower limbs [54].

Trybulski et al. (2024) [17] in myotonometric measurements of the rectus femoris and vastus medialis performed in athletes showed high and very high repeatability of two measurements performed with an interval of 30 s by two evaluators. Depending on the parameter and the analyzed muscle group, the ICC value ranged from 0.74 to 0.99, except for the study of the stiffness of the vastus medialis (ICC = 0.08) [18].

In this project, the authors attempted to verify the repeatability of the examination of physical characteristics of muscles in children aged 0–3 months using the Myoton Pro device. These tests were among the initial assessments conducted on a group of infants, aiming to minimize potential measurement bias and lessen the impact of confounding clinical factors—such as neurological impairments, altered muscle tone, or systemic conditions that could affect tissue properties. Therefore, the study exclusively included healthy, full-term infants who had no diagnosed developmental, neurological, or genetic disorders. Selecting a homogeneous and clinically stable population was crucial to ensure that the variability in the obtained parameters accurately reflected true measurement repeatability, rather than pathological changes in muscle structure or function. For the validation of diagnostic assays, the samples used in repeatability and reproducibility studies must be homogeneous and stable to guarantee that any variability is attributable to the test itself, rather than the sample being analyzed [55]. This approach may impose constraints on the external validity of the findings presented. Specifically, the documented stability and repeatability of myotonometric parameters observed within a healthy population cannot be readily extrapolated to infants at risk of developmental disorders, as the neuromuscular characteristics in this demographic may significantly diverge. Moreover, the clinical applicability of the findings remains constrained, as the diagnostic potential of myotonometric measurements necessitates validation within populations exhibiting altered neuromuscular function. In such cohorts, variability may be more pronounced, and the behavior of elastoplastic muscle parameters may manifest distinct patterns, potentially impacting both the reliability of measurements and their interpretability. Consequently, while this study offers critical preliminary data under controlled conditions, future research should aim to extend these findings to broader and more clinically pertinent populations. Specifically, investigations that include infants at risk for neurodevelopmental disorders, along with comparative analyses involving established clinical assessment tools, will be vital for elucidating the true diagnostic and prognostic value of myotonometry in early infancy.

The basic criterion for assessing the clinical and research usefulness of a measurement is its reliability. Reliability is defined as the level to which the measure is free from random errors. It is quantified by the degree to which measurements are consistent (consistent/stable) and repeatable [11,38,41,56]. Research in both adult [15] and older pediatric [57] populations has shown impressive measurement repeatability (ICC ≥ 0.9), both at rest and under load, underscoring the tool’s effectiveness in evaluating lower limb muscles. This device allows for the assessment of muscle tone, stiffness, muscle elasticity, mechanical stress relaxation time, and ratio relaxation and deformation time [58].

Lukáš et al. (2023) [10] analyzed upper and lower limb muscles, including the biceps, triceps, and gastrocnemius, to better quantify myotonia and muscle stiffness [21]. Carroll et al. (2021) [58] focused on the calf complex, particularly the gastrocnemius, to investigate whether massage interventions could reduce passive stiffness and support muscle length management in ambulant boys [59]. In spinal muscular atrophy types I–II, Kutlutürk Yıkılmaz et al. (2024) [11] measured the biceps brachii, triceps brachii, rectus femoris, and gastrocnemius to establish intra- and inter-rater reliability of MyotonPRO assessments for clinical follow-up [57]. A recent systematic review has confirmed the reliability of myotonometric measures across more than 30 muscles, reinforcing their suitability for evaluating both the upper and lower limbs in neuromuscular disease research [60].

Our own studies, including three measurements of viscoelastic parameters of the lateral head of the gastrocnemius muscle performed in children on the 1st–3rd day of life, 6th week, and 12th week of life, showed significant differences in repeatability. In the first days of the child’s life, the repeatability of measurements of all analyzed features can be described as low or moderate. ICC values were in the range of 0.28–0.65. Three measurements performed in the 6th week of life showed statistically significant, moderate, or high repeatability of stiffness, elasticity, muscle tone, relaxation time, and creep (ICC 0.59–0.75). Studies performed in the 12th week showed a significant increase in repeatability. The repeatability of the measurements of muscle stiffness, muscle tone, relaxation time, and the ratio of relaxation time to deformation time (creep) estimated by the intraclass correlation coefficient value can be defined as high and very high (ICC 0.77–0.90), and flexibility as moderate (ICC = 0.56). The increase in the repeatability of measurements of physical muscle characteristics with the development of the child, especially muscle stiffness and muscle tone, may result from changes occurring in the neuromuscular system during the maturation of the newborn [61]. Histopathological studies prove changes occurring in the fetal and neonatal muscle concerning the withdrawal of polyneuronal innervation and changes occurring in the structure and function of the acetylcholine receptor and the acetylcholine unit itself. The characteristic structure of the neuromuscular system in the neonatal period determines spontaneous fetal movements, which are necessary for the proper development of the neuromuscular system [61,62,63].

Measurements performed on newborns and infants are extremely difficult to perform due to the lack of logical contact with the examined person, the child’s mobility, and the small size of the areas examined. Finding objective methods for assessing the state of the musculoskeletal system in infants with confirmed measurement reliability analyses is extremely important in the diagnostic process and monitoring of therapeutic processes [48].

Early recognition of muscle tone disorders is vital for ensuring timely intervention. However, it is important to consider that biomechanical measurements taken before 6 weeks of age may have limited repeatability due to tissue immaturity and variability in physiological responses. To enhance diagnostic accuracy and data quality, it may be beneficial to conduct assessments between 6 and 12 weeks of age, as this timeframe often yields more reliable results. By incorporating objective measurement methods, such as myotonometry, alongside clinical qualitative assessments, we may significantly improve the overall diagnostic process. This balanced approach not only may support early intervention but also may have the potential to ensure the data collected is robust and reliable.

## 5. Conclusions

The repeatability of myotonometric measurements in infants aged 1–3 days appears to be low to moderate, whereas measurements obtained at 6 weeks demonstrate moderate to high repeatability across most viscoelastic parameters. At 12 weeks of age, repeatability may be considered high; however, these findings should be interpreted with caution due to the limited sample size at this time point.

Overall, myotonometric measurements using the Myoton Pro device may provide a potentially useful source of objective data, particularly in infants older than 6 weeks. Nevertheless, the present findings should be viewed as preliminary. Key limitations, including the relatively small sample size, substantial attrition during follow-up, and the inclusion of only healthy participants, may restrict the generalizability and clinical applicability of the results. Further research involving larger cohorts and clinically diverse populations is required to confirm these observations.

This study should be considered a preliminary pilot investigation emphasizing repeatability, rather than as conclusive evidence of clinical applicability. Subsequent research is essential to validate these findings in relation to established clinical assessment tools, such as the General Movements Assessment (GMA) and the Hammersmith Infant Neurological Examination (HINE). Furthermore, it is imperative to expand the scope of the investigation to include populations that are at risk for developmental disorders.

### Limitations of the Study

The main limitation of the study is the decreasing number of subjects over time. The first study was completed by 49 children, the third by 12. As the dropout effect remains strong, 12-week results should be interpreted with caution. This is a common problem in studies of healthy children. Study 1 was performed in the hospital after the child was born. Children had to come specially for studies 2 and 3. Healthy children do not require additional medical consultations or therapy, so parents often skip the planned visit despite earlier declarations. Conducting research in infant populations is time-consuming and requires strong collaboration with parents. Their involvement is essential to ensure appropriate preparation and successful completion of study procedures. Inter-rater reliability was not assessed in this study, as the design focused on test–retest reliability. This should be addressed in future studies. This study should be considered a pilot (exploratory) study due to the relatively small sample size, which may limit the precision and stability of the ICC estimates and restrict the generalizability of the findings.

## Figures and Tables

**Table 1:** Mechanical properties of the triceps calf muscle on the 1st–3rd day of a child’s life.

Age 1–3 Day									
	Measurement 1			Measurement 2			Measurement 3		
Variable	Mean	SD	SE	Mean	SD	SE	Mean	SD	SE
F	16.56	3.08	0.44	16.74	3.13	0.45	16.81	3.43	0.49
S	284.33	61.57	8.89	295.20	71.27	10.18	292.11	79.89	11.41
D	1.85	0.60	0.09	1.93	0.57	0.08	1.94	0.53	0.08
R	19.12	3.64	0.52	18.53	419	0.60	18.72	3.85	0.55
C	1.17	0.22	0.03	1.13	0.25	0.04	1.16	0.27	0.04

F—muscle tone, S—stiffness, D—elasticity, R—relaxation, C—creep, SD—standard deviation, SE—standard error.

**Table 2:** Agreement of measurements determined by the intraclass correlation coefficient (ICC) together with the confidence interval (±95%) obtained in the measurements of mechanical properties of the triceps calf muscle in subsequent measurements (1, 2, 3) on days 1–3 of life.

		Age 1–3 Day					
Variable	ICC (2,1) for Single Measurement	−95% CI	+95% CI	SEM	MDC_95_	Statistics F	*p*-Value
F	0.49	0.31	0.64	2.20	6.10	3.78	<0.0001 *
S	0.28	0.11	0.47	52.24	144.81	2.18	0.0007 *
D	0.54	0.38	0.69	0.41	1.13	4.51	<0.0001 *
R	0.65	0.51	0.77	2.15	5.97	6.66	<0.0001 *
C	0.58	0.42	0.71	0.14	0.40	5.06	<0.0001 *

F—muscle tone, S—stiffness, D—elasticity, R—relaxation, C—creep, SEM—Standard Error of Measurement, MDC_95_—Minimal Detectable Change * *p* < 0.05.

**Table 3:** Mechanical properties of the triceps calf muscle in the 6th week of a child’s life.

Age 6 Weeks									
	Measurement 1			Measurement 2			Measurement 3		
Variable	Mean	SD	SE	Mean	SD	SE	Mean	SD	SE
F	15.72	2.19	0.53	15.31	2.43	0.59	15.39	2.14	0.52
S	278.47	77.48	18.79	265.41	58.50	14.19	268.00	59.78	14.50
D	1.49	0.35	0.09	1.47	0.32	0.08	1.48	0.30	0.07
R	18.42	3.87	0.94	20.32	3.89	0.94	19.80	3.20	0.78
C	1.14	0.22	0.05	1.24	0.23	0.06	1.21	0.22	0.05

F—muscle tone, S—stiffness, D—elasticity, R—relaxation, C—creep, SD—standard deviation, SE—standard error.

**Table 4:** Agreement of measurements determined by the intraclass correlation coefficient (ICC) together with the confidence interval (±95%) obtained in the measurements of mechanical properties of the triceps calf muscle in consecutive measurements (1, 2, 3) at 6 weeks of age.

		Age 6 Weeks					
Variable	ICC (2,1) for Single Measurement	−95% CI	+95% CI	SEM	MDC_95_	Statistics F	*p*-Value
F	0.69	0.45	0.86	1.22	3.38	7.48	<0.0001 *
S	0.74	0.52	0.89	39.51	109.51	9.45	<0.0001 *
D	0.75	0.53	0.89	0.18	0.49	9.56	<0.0001 *
R	0.68	0.43	0.85	2.19	6.07	7.64	<0.0001 *
C	0.59	0.33	0.81	0.14	0.39	5.82	<0.0001 *

F—muscle tone, S—stiffness, D—elasticity, R—relaxation, C—creep, SEM—Standard Error of Measurement, MDC_95_—Minimal Detectable Change * *p* < 0.05.

**Table 5:** Mechanical properties of the triceps calf muscle in the 12th week of the child’s life.

Age 12 Weeks									
	Measurement 1			Measurement 2			Measurement 3		
Variable	Mean	SD	SE	Mean	SD	SE	Mean	SD	SE
F	14.12	1.79	0.54	14.04	1.94	0.58	14.05	2.17	0.66
S	251.09	38.38	11.57	242.36	53.08	16.00	252.00	54.35	16.39
D	1.52	0.23	0.07	1.37	0.26	0.08	1.35	0.27	0.08
R	22.37	4.57	1.38	22.10	3.87	1.17	21.84	4.03	1.22
C	1.36	0.28	0.09	1.32	0.21	0.06	1.33	0.23	0.07

F—muscle tone, S—stiffness, D—elasticity, R—relaxation, C—creep, SD—standard deviation, SE—standard error.

**Table 6:** Agreement of measurements determined by the intraclass correlation coefficient (ICC) together with the confidence interval (±95%) obtained in the measurements of mechanical properties of the triceps calf muscle in consecutive measurements (1, 2, 3) at 12 weeks of age.

		Age 12 Weeks					
Variable	ICC (2,1) for Single Measurement	−95% CI	+95% CI	SEM	MDC_95_	Statistics F	*p*-Value
F	0.90	0.76	0.97	0.57	1.57	26.45	<0.0001 *
S	0.77	0.50	0.92	18.41	51.02	10.59	<0.0001 *
D	0.56	0.21	0.83	0.15	0.42	5.60	0.0005 *
R	0.82	0.58	0.94	1.94	5.37	13.33	<0.0001 *
C	0.81	0.58	0.94	0.12	0.34	13.56	<0.0001 *

F—muscle tone, S—stiffness, D—elasticity, R—relaxation, C—creep, SEM—Standard Error of Measurement, MDC_95_—Minimal Detectable Change * *p* < 0.05.

## Data Availability

The data that support the findings of this study are not openly available due to reasons of sensitivity and are available from the corresponding author upon reasonable request. Data are located in controlled-access data storage. The dataset analyzed in the present study constitutes part of a larger, ongoing research project. For this reason, and to ensure consistency of analyses and data integrity, the data are currently available upon reasonable request.

## Supplementary Materials

The following supporting information can be downloaded at: https://www.mdpi.com/article/10.3390/jcm15103699/s1, STROBE_checklist; File S1: Ryc. 3 Bland-Altman 1-3 days; File S2: Ryc. 4 Bland-Altman 6 weeks; File S3: Ryc. 5 Bland-Altman 12 weeks.
