# Supplementary material for: Repeatability of Myotonometric Muscle Measurements in Infants Aged 0–3 Months: Toward an Objective Tool Supporting Early Motor Assessment
Source: J Clin Med. 2026 May 11;15(10):3699. doi: 10.3390/jcm15103699 (PMC13206822; doi:10.3390/jcm15103699)
Supplement: Supplementary file 1 [file jcm-15-03699-s001.zip › File S1--Ryc. 3 Bland-Altman 1-3 days.pdf]

Ryc. 3. Bland–Altman plots for measurements obtained in infants during the first 1–3 days of life.(3.1-F, 3.2-S, 3.3-D, 3.4-R, 3.5-C)

3.1

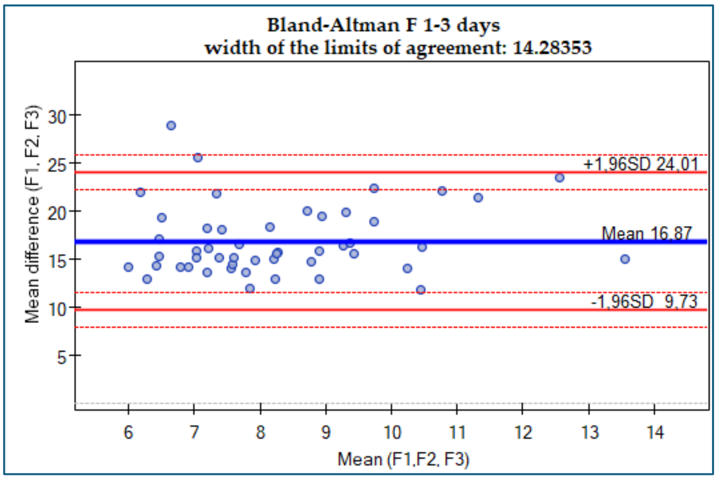

3.2

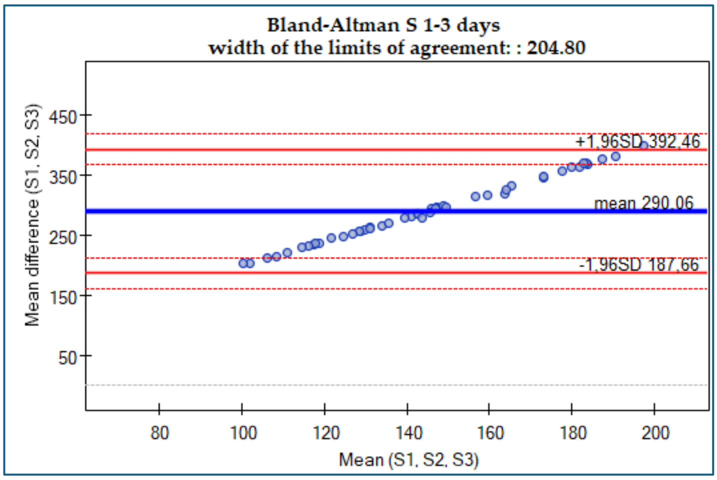

3.3.

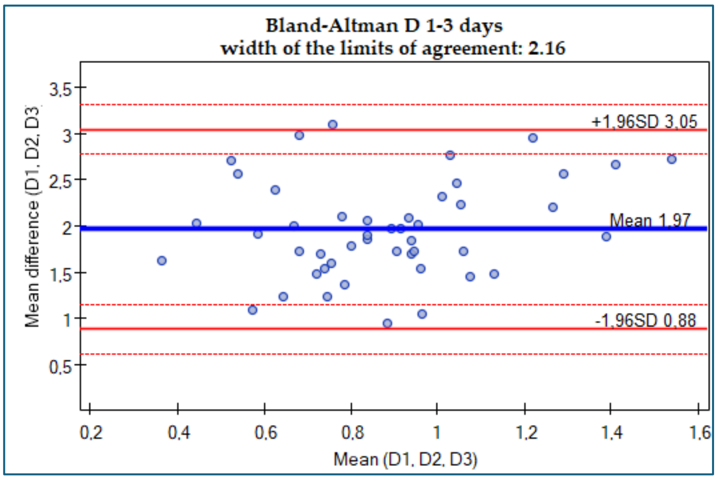

3.4

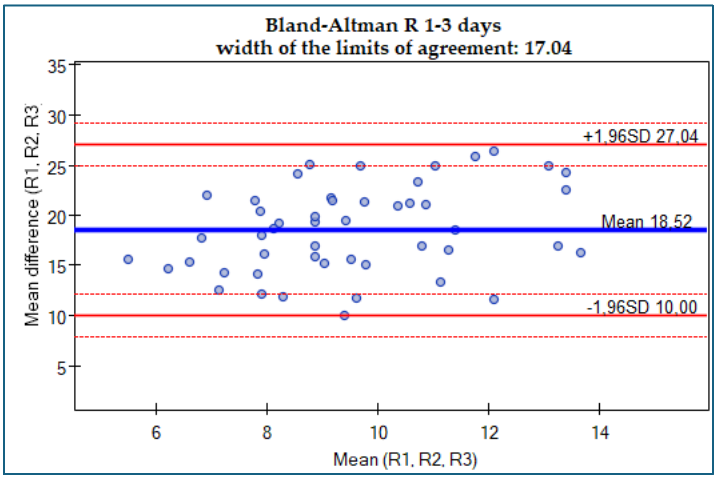
Bland-Altman R 1-3 days
width of the limits of agreement: 17.04
+1,96SD 27,04
Mean 18,52
-1,96SD 10,00
Mean difference (R1, R2, R3)
Mean (R1, R2, R3)
35
30
25
20
15
10
5
6
8
10
12
14

3.5

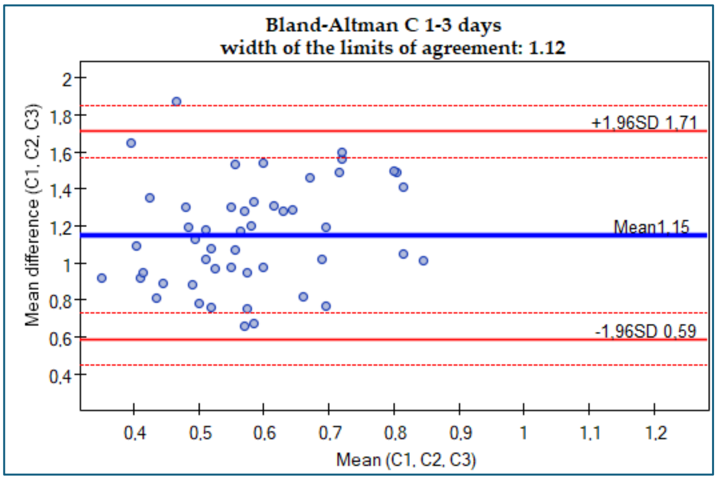
Bland-Altman C 1-3 days
width of the limits of agreement: 1.12
+1,96SD 1,71
Mean1,15
-1,96SD 0,59
Mean difference (C1, C2, C3)
Mean (C1, C2, C3)
2
1,8
1,6
1,4
1,2
1
0,8
0,6
0,4
0,4
0,5
0,6
0,7
0,8
0,9
1
1,1
1,2
