# Supplementary figures and images for: Repeatability of Myotonometric Muscle Measurements in Infants Aged 0–3 Months: Toward an Objective Tool Supporting Early Motor Assessment
Source: J Clin Med. 2026 May 11;15(10):3699. doi: 10.3390/jcm15103699 (PMC13206822; doi:10.3390/jcm15103699)

Ryc. 4. Bland–Altman plots for measurements performed at 6 weeks of life in infants. (4.1- F, 4.2-S, 4.3-D, 4.4-R, 4.5-C)

4.1

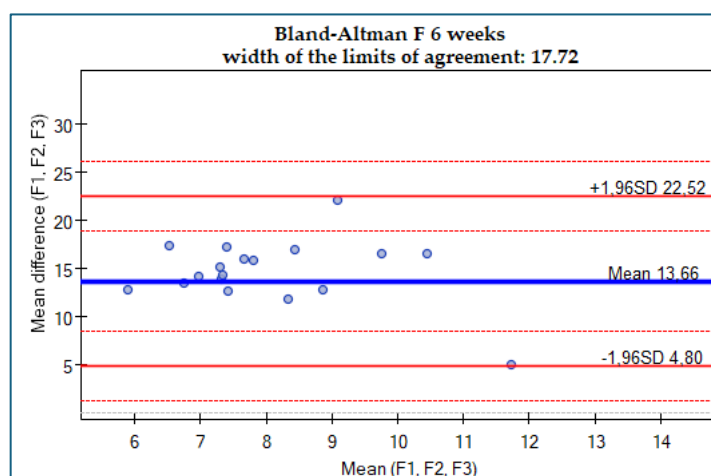

4.2

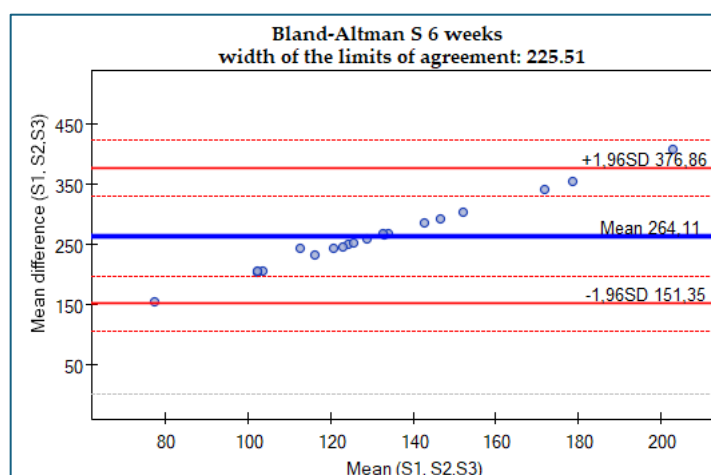

4.3.

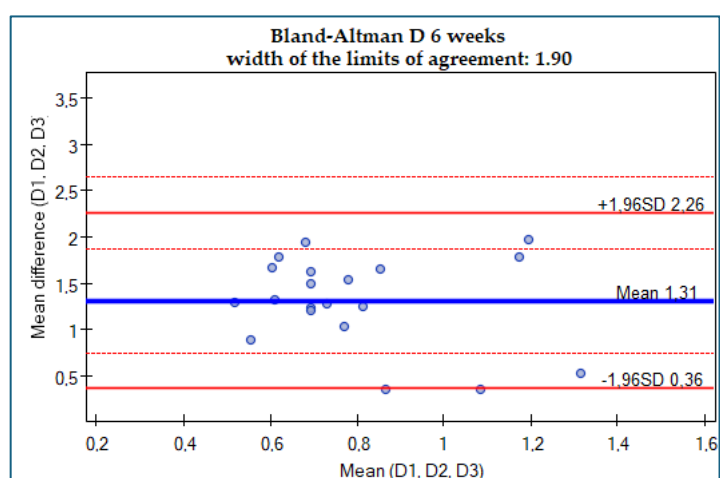

4.4

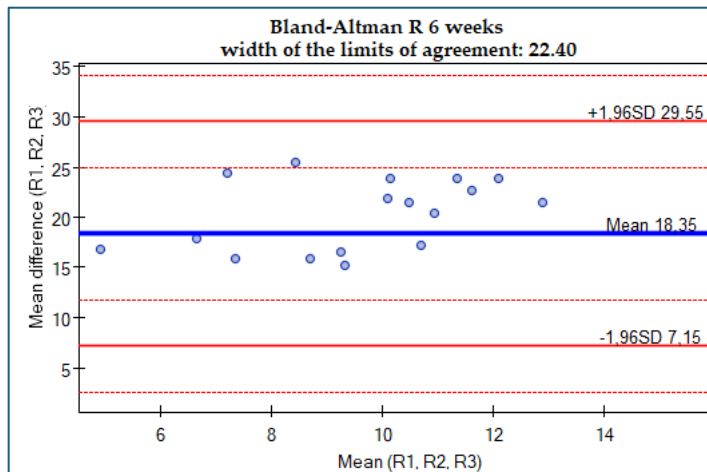

4.5

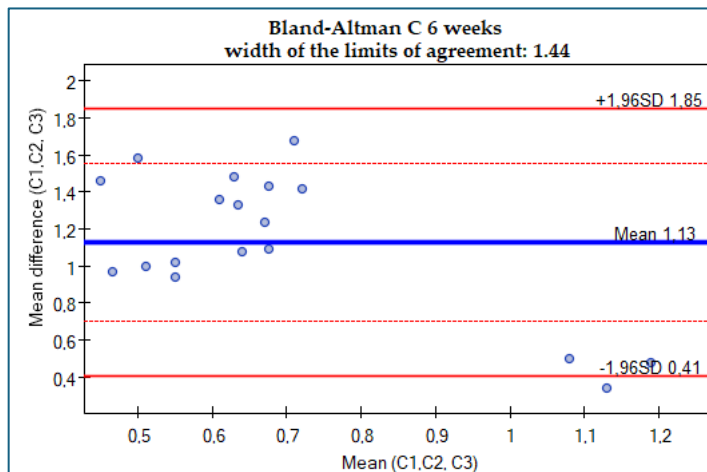

Supplement: Supplementary file 1 [file jcm-15-03699-s001.zip › File S2--Ryc. 4 Bland-Altman 6 weeks.pdf]

Ryc. 5. Bland–Altman plots for measurements performed at 12 weeks of life in infants. (5.1- F, 5.2-S, 5.3-D, 5.4-R, 5.5-C)

5.1

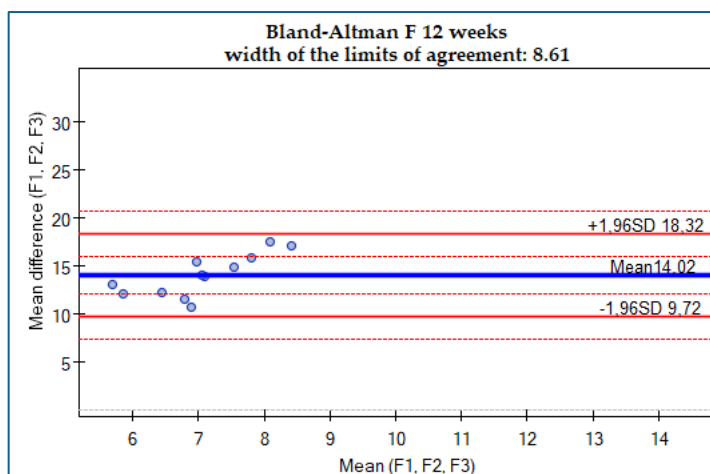

5.2

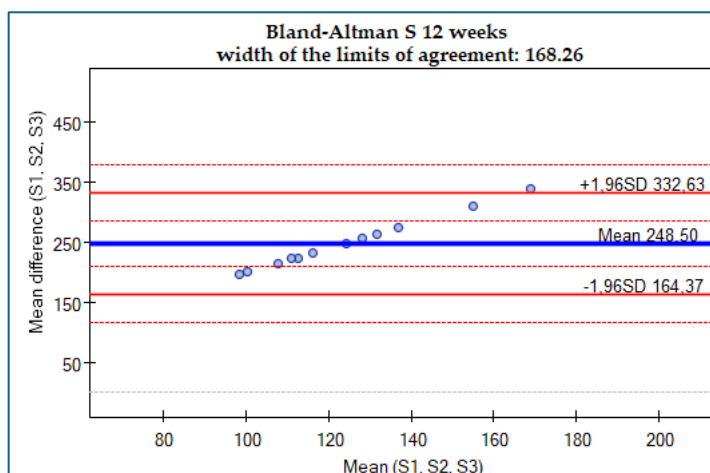

5.3.

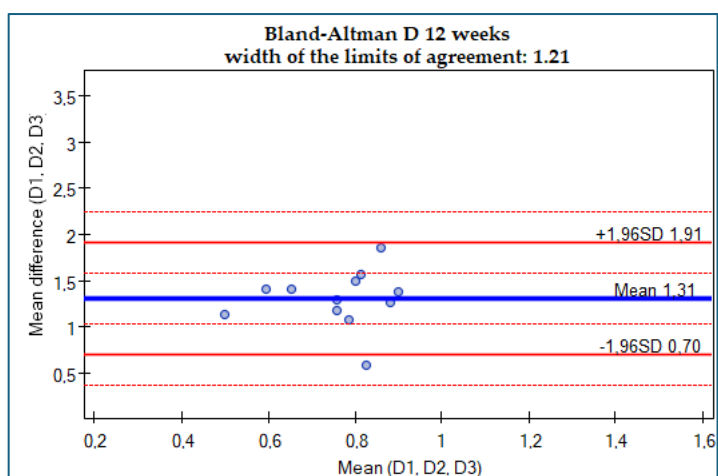

5.4

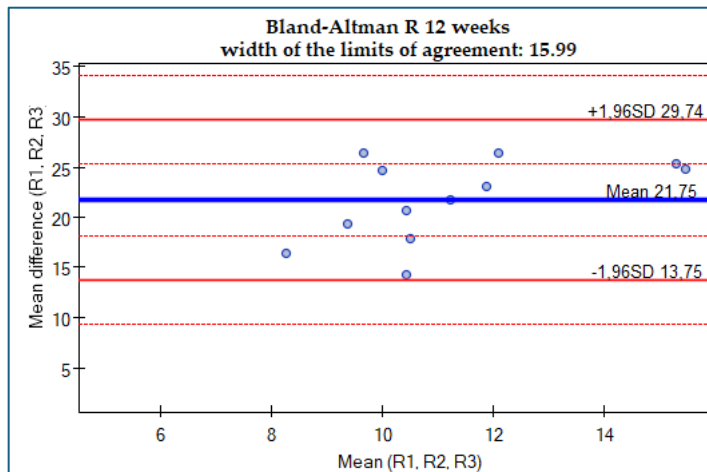

5.5

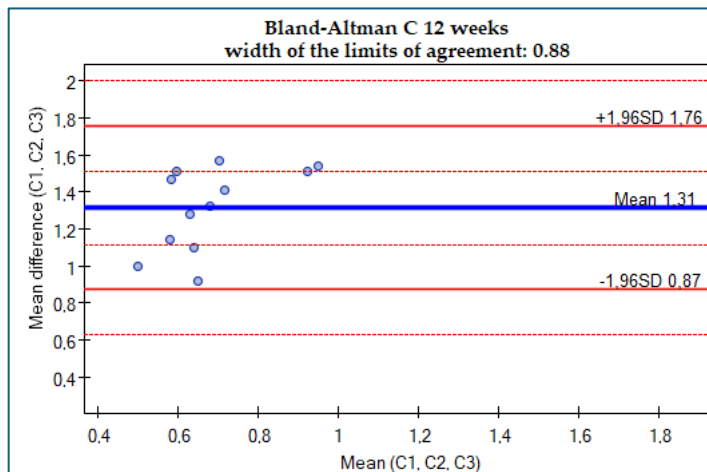

Supplement: Supplementary file 1 [file jcm-15-03699-s001.zip › File S3--Ryc. 5 Bland-Altman 12 weeks.pdf]
